# Could Vitamin D3 Deficiency Influence Malocclusion Development?

**DOI:** 10.3390/nu13062122

**Published:** 2021-06-21

**Authors:** Anna Leszczyszyn, Sylwia Hnitecka, Marzena Dominiak

**Affiliations:** 1 Oral Surgery Department, Wroclaw Medical University, 50-425 Wroclaw, Poland; lejerzyna@gmail.com (A.L.); marzena.dominiak@umed.wroc.pl (M.D.); 2 Maxillofacial Surgery Department, Wroclaw Medical University, 50-556 Wroclaw, Poland

**Keywords:** vitamin D, malocclusion, maxillary constriction, orthodontics, vitamin D deficiency

## Abstract

The abnormal growth of the craniofacial bone leads to skeletal and dental defects, which result in the presence of malocclusions. Not all causes of malocclusion have been explained. In the development of skeletal abnormalities, attention is paid to general deficiencies, including of vitamin D3 (VD3), which causes rickets. Its chronic deficiency may contribute to skeletal malocclusion. The aim of the study was to assess the impact of VD3 deficiency on the development of malocclusions. The examination consisted of a medical interview, oral examination, an alginate impression and radiological imaging, orthodontic assessment, and taking a venous blood sample for VD3 level testing. In about 42.1% of patients, the presence of a skeletal defect was found, and in 46.5% of patients, dentoalveolar malocclusion. The most common defect was transverse constriction of the maxilla with a narrow upper arch (30.7%). The concentration of vitamin 25 (OH) D in the study group was on average 23.6 ± 10.5 (ng/mL). VD3 deficiency was found in 86 subjects (75.4%). Our research showed that VD3 deficiency could be one of an important factor influencing maxillary development. Patients had a greater risk of a narrowed upper arch (OR = 4.94), crowding (OR = 4.94) and crossbite (OR = 6.16). Thus, there was a link between the deficiency of this hormone and the underdevelopment of the maxilla.

## 1. Introduction

The growth and development of jawbones are influenced by various genetic and environmental factors, dysfunctions, and parafunctions, such as thumb sucking and mouth breathing [1,2]. The insufficient or excessive growth of the craniofacial bone leads to skeletal and dental defects, which, in turn, result in the presence of malocclusions. According to the current Orlik-Grzybowska classification, malocclusion is assessed in the sagittal, frontal, and horizontal planes [2], and divided into vertical, horizontal, and transverse. There are skeletal, dental, and mixed defects.

To date, not all causes of malocclusion have been fully explained. Moyers et al. distinguished six categories of malocclusions: hereditary, developmental of unknown origin, trauma, physical factors, habits, and diseases [2,3]. Karłowska, on the other hand, divided the causes of such abnormalities into general (heredity, endocrine disorders, systemic diseases, extrinsic factors, e.g., improper position of the fetus, mechanical pressure caused by e.g., tumor, avitaminosis, effects of drugs, etc.) and local (dysfunctions, parafunctions, caries, injuries, etc.) [2].

In the development of skeletal abnormalities, attention is often paid to general deficiencies, including vitamin D3 deficiency, which causes rickets in growing people. The deficiency affects the jawbone and might be involved in malocclusion development. This vitamin is endogenously produced in the body and provided in the diet (fatty fish, eggs, dairy products, nuts). The synthesis of this hormone by photolysis in the skin takes place under the influence of UVB radiation. Environmental and geographic factors, e.g., latitude, season, weather, the angle of incidence of the sun rays, cloud covering, clothing (the degree of covering the body with clothes), and time spent in the sunlight, play an important role in this process [4]. This hormone exerts a pleiotropic effect, influencing the calcium–phosphate balance, and the maintenance of bone function and metabolism [5,6]. Moreover, medicine proves its pleiotropic effect, and thus its influence on both bone structure (it being a risk factor of osteoporosis, osteomalacia, and fractures) and on the development of cardiovascular diseases, obesity, hypocalcemia, cancer, diabetes, depression, and autoimmune diseases [7]. Chronic deficiency in children causes rickets, leading to skeletal abnormalities, a short stature, and delayed skeletal development [7].

Deficiency in this vitamin is global, even in highly sunlit regions [8]. A blood concentration below 30 ng/mL (75 nmol/L) is considered to be a deficiency [9]. The role of vitamin D3 in oral and maxillofacial sciences is significantly less discussed. Its significant impact on oral health was demonstrated, showing that its deficiency increases the risk of periodontitis, alveolar bone loss, periimplantitis, caries, oral cancer, and osteonecrosis due to bisphosphonate treatment [10,11,12,13]. However, its unequivocal influence on the development of malocclusions has not been assessed so far. Its chronic deficiency could be a significant factor modulating the development of skeletal defects in children, especially such as the narrowing and shortening of the arches, maxillary and mandibular narrowing, micrognathia, microgenia, and retrogenia.

Malocclusions are often complex, so the disorder usually occurs in several planes and not as an isolated defect [1].

For example, a narrow maxilla with a narrow upper dental arch in relation to the width of the face, a high arched palate, a crossbite, a palatal tilt of the upper teeth, and crowding are the causes of difficult functioning [2]. Common dark cheek tunnels and crowding in the upper arch negatively affect the aesthetic aspect. Patients also complain of the need to breathe with their mouths due to the obstruction of the upper respiratory tract, allergies, frequent colds, and tonsil hypertrophy. Similarly, the shortening of the dental arch as a result of anteroposterior maxillary hypoplasia is characterized in clinical examination by a deep nasolabial fold, the shortening of the dental arch, and the crowding of teeth [3]. In some cases, jawbone development is inhibited in all three dimensions, resulting in microgenia or micrognathia [2]. Micrognathia is accompanied by pseudo-occlusion with a bilateral crossbite. It is often associated with clefts, and genetic syndromes such as Crouzon and Apert syndrome. Morphological hindquarters result from the inhibition of the front growth of the mandible and other changes in its structure. The mandibular ramus is usually shortened. The chin is set back, often accompanied by the elongation of the lower part of the face. It is a developmental defect that intensifies as a result of past rickets [1,3]. These defects significantly negatively affect both functional and aesthetic aspects, and require specialized multidisciplinary treatment.

The aim of the study was to prospectively assess the impact of vitamin D deficiency on the development of malocclusions.

## 2. Materials and Methods

### 2.1. Research Group

A prospective observational study was conducted in a randomly selected group of patients from a private dental practice in Wrocław in 2017–2018. Analysis included patients of both sexes, Caucasian above 18 years of age, who had come for treatment for various reasons (usually with caries or with orthodontic problems). The study excluded people currently undergoing oncology treatment; treated with bisphosphonates; denosumab and angiogenesis inhibitors; pregnant women; patients with severe gastrointestinal diseases with chronic diarrhea; after extensive resections in the gastrointestinal tract, especially in the small intestine; patients with past facial trauma; active degenerative joint disease; developmental abnormalities, such as condylar hypo- or hyperplasia; and those with a history of surgical jaw treatment. For patients who had undergone orthodontic treatment, the initial malocclusion was described, as the patients had been treated in this dental practice from the beginning, and we had access to all necessary clinical data (patients who were included in this study had been orthodontically treated for a maximum of 1 year (teeth alignment only). All participants or their legal guardians were acquainted with the subject of the study and gave their informed consent to participate in the project.

### 2.2. Research Components

The examination consisted of three parts: (1) medical interview; (2) oral examination with an alginate impression and radiological imaging (panoramic and cephalometric radiograph), and orthodontic assessment; and (3) taking a venous blood sample for vitamin D3 level testing.

The interview included demographic data (age, sex, height, body weight), habits (smoking, alcohol consumption, diet, last summer vacation in the sunlight, vitamin D3 supplementation), accompanying systemic diseases, medications, and past orthodontic treatment.

A detailed examination of the oral cavity assessed canine and Angle’s class [2] on both sides. If the first molar or canine was missing, the class on this side was not assessed, vertical and horizontal overbite (in millimeters), crowding on the three-point Hotz scale (1970) in the maxilla and the mandible: 1st degree—no space for half of the incisal tooth, 2nd degree—no space for one and a half incisors, 3rd degree—no space for 2 or more incisors. The second and third degrees indicated extraction treatment needs. Malocclusion (in the sagittal, horizontal, and orbital planes according to Karłowska [2]) was also evaluated.

Each patient underwent an alginate impression to prepare a plaster diagnostic model for analysis, an intraoral radiograph showing the upper and lower incisors, and volumetric tomography of the maxillary and mandibular regions using a Carestream^®^ device (Carestream Health, New York, USA).

In the periodontal aspect, the depth of the periodontal pockets with the use of WHO 621 probe and clinical attachment level or loss (CAL) were assessed. When the gingival margin was coronal to the cementoenamel junction (CEJ), the CAL was calculated by subtracting the gingival margin level from the probing depth (3 mm as a physiological value). The classification of Periodontal and Peri-Implant Diseases and Conditions (Chicago, November 2017, American Academy of Periodontology (AAP) and the European Federation of Periodontology (EFP)) was used to assess the occurrence and severity of periodontitis.

Soft tissue was also assessed. In the case of gingival recession, the advancement was determined according to Miller’s classification [14].

Laboratory tests of venous blood collected from the elbow fossa were also performed. The blood sample was collected into a special tube, stored in a refrigerator after collection, and then, every 2 h, collectively transported vertically in a portable refrigerator to a certified Dialab laboratory in Wrocław. The study gave concentration of vitamin 25 (OH) D metabolite, determined by the immunochemical method. Vitamin 1.25 (OH) 2 was determined at the Synlab laboratory in Warsaw using the enzyme immunoassay (EIA) method.

Reference values were: vitamin 25 (OH) D: 20.0 ng/mL, deficit; 20–30 ng/mL, too low level; 30–50 ng/mL, optimal level; 50–100 ng/mL, too high level; and ≥100 ng/mL, toxic level.

### 2.3. Statistical Analysis

For quantitative variables, basic descriptive statistics were calculated: means (M), standard deviations (SD), medians (Me), lower (Q1) and upper (Q3) quartiles, and extreme values: smallest (Min) and largest (Max). The consistency of empirical distributions of quantitative continuous variables with theoretical normal distribution was established using the Shapiro–Wilk test, and the homogeneity of variance using the Levene and Brown–Forsythe tests. Nominal qualitative and ordinal variables are presented in multiway (cross) tables in the form of count (*n*) and proportion (%). The significance of differences in the mean values of variables with a distribution close to normal in the two groups was verified with the Student’s *t*-test. If the empirical distribution in any of the groups differed from the normal, the nonparametric Mann–Whitney U test was used. In the case of a larger number of groups, analysis of variance (ANOVA) or the Kruskal–Wallis test was used. The strength and direction of the relationship between the two quantitative variables was estimated by calculating the Pearson correlation coefficient r. Data were analyzed with Statistica v. 13 (StatSoft Inc, Tulsa, OK, USA). Statistical significance for all statistical tests was set at *p* < 0.05.

The research was performed in accordance with the Declaration of Helsinki and was approved by the ethics committee of Wrocław Medical University (KB-442/2017) in 2017.

## 3. Results

Analysis included 114 patients (53 men and 61 women) aged 18–50 years (mean 36.5 ± 11.8 years) who had completed their medical interview, participated in the clinical examination, and donated a blood sample for laboratory tests.

In the studied group of patients, the majority of people denied vitamin D3 supplementation (73.6% *n* = 84). Only nearly one-quarter of all respondents declared taking this vitamin, while the patients took small doses (500–2000 IU per day) of vitamin D3 alone, without calcium and vitamin K2.

### 3.1. General Information and Habits

Exposure in the sunlight during the last 6 months was declared by 21.9% of patients. The profile of the examined people could be described as generally healthy, conscious people, with a relatively high quality of life; hence, the accompanying systemic diseases did not exceed around 12% (autoimmune, 13.2%; gastrointestinal, 8.8%; cardiovascular, 4.4%; metabolic, 0.9%). Proper hygienic habits, that is, using a toothbrush with an appropriate technique for more than 2 min at least twice a day and regular use of an irrigator or dental floss, were found in 35.1% of respondents. In total, 40 people (35.1%) had the habit of grinding their teeth.

### 3.2. Skeletal and Dentoalveolar Status

In about 42.1% of patients, the presence of a skeletal defect was found. In 46.5% of patients, dentoalveolar malocclusion was found. Most of the patients presented Angle and Canine Class I. The most common defect was transverse constriction of the maxilla with a narrow upper arch (30.7%). Moreover, 85 patients had crowding, 50 in the lower jaw and 35 in the upper jaw. In addition, 28.1% had periodontal disease with the presence of pathological periodontal pockets. In turn, Class I recessions according to Miller were found in 22.8% of patients, second class in 15.8% of patients, and third class in 17.5% of patients Table 1. Tooth abfractions and attritions were found in near one-half of the cases (50.5%).

### 3.3. Vitamin D Serum Level

The concentration of vitamin 25 (OH) D in the study group ranged from 7.8 to 57 (ng/mL), with an average of 23.6 ± 10.5 (ng/mL). In accordance with the adopted refrain values, its deficiency was found in 86 subjects (75.4%; level up to 30 ng/mL; Table 2).

In the group of patients with optimal and overstated vitamin D3 levels, the assessment of bite correctness analyzed together (Angle’s class on the left and right) was significantly better than that in patients with deficiency or undervalued D3 concentration (*p* < 0.05). The relationships between skeletal and dental malocclusion of canine classification, the difference in favor of patients with normal D3 concentration remained at the border of statistical significance (*p* = 0.087; Table 3).

Overall risk factors for vitamin D deficiency are defined in Table 3.

In the group of patients of up to 28 years of age, vitamin D deficiency was more frequent. The deficit was more common in men. People with a deficiency of vitamin D more often did not supplement vitamin D3. Vitamin D3 deficiency was more common in people with loss of connective tissue attachment and pathological periodontal pockets. It was shown that vitamin D deficiency negatively influences the maxillary development. These patients were shown to be at greater risk of narrowed upper arch (OR = 4.94), crowdings in upper arch (OR = 4.94) and crossbite (OR = 6.16) Table 4.

## 4. Discussion

The positive effect of vitamin D on bone health has been known for a long time. It is believed to be closer to hormones than to vitamins because it is endogenously produced, and its action is not only mediating calcium–phosphate homeostasis. Vitamin D3 deficiency is a global issue that affects both healthy people and those with comorbidities, regardless of latitude, age, gender, and race [15,16]. Most newborns are not exposed to sunlight, often having vitamin D deficiency, with 25 (OH) D levels < 50 nmol/L [17]. This may indicate that vitamin D deficiency already occurs during pregnancy. Other authors believe that prenatal vitamin D status appears to affect postnatal mineral homeostasis and may influence growth. Postnatal vitamin D status is fundamental to mineral homeostasis and may affect subsequent bone mass [18]. The effect of vitamin D3 levels on dentogingival status has not yet been fully elucidated, and the number of scientific publications on this topic is limited.

Most available studies concern the assessment of the effect of vitamin D3 on gingivitis and periodontitis. A study by Krall et al. on a group of 145 people over 65 years of age showed that supplementation with calcium and vitamin D3 reduces the risk of tooth loss in the elderly [19]. Zahn et al. also noted significant correlation between vitamin D deficiency and tooth loss [20]. In the nonsupplementing group that we studied, bruxism was observed in 38.8% of patients, and in 54.1%, various types of teeth overload (attrition, abfractions). In this group, the mean value of vitamin D was found to be at the level of 20.25 ± 7.7 ng/mL (min–max: 15.2–25.5 ng/mL), so there was a deficit in accordance with the adopted reference ranges. Research by Singleton et al., and Schroth et al. indicated that prenatal vitamin D levels may influence the primary dentition and the development of early childhood caries (ECC), so improving vitamin D status in pregnant women might affect ECC rates in their infants [21,22].

In orthodontics, vitamin D deficiency may affect the slower movement of teeth under the influence of orthodontic forces, which was confirmed in several studies [23,24]. On the basis of studies on rats, the authors concluded that the use of calcitriol may promote the reconstruction of the surrounding tissue after orthodontic treatment. Proper vitamin D3 levels during orthodontic treatment may affect more effective bone remodeling, especially in the elderly [23]. However, in the studies of Tehranchi and colleagues in 34 orthodontically treated patients with the use of fixed appliances, levels of vitamin D3 were not associated with external root resorption [24]. Although no such correlation was found, the researchers stressed that research in this area should be expanded, as there are few scientific reports on the subject.

Our research did not evaluate the influence of vitamin D levels on the course of orthodontic treatment. As it was a prospective observational study, only vitamin D levels were associated with malocclusion or dental-defect occurrence. Retrospective analysis was performed on patients with completed maxilla transverse growth (after claw eruption). Patients with low vitamin D levels either did not supplement it at all, or did so in small doses that did not significantly affect the levels. Some trends were observed that, due to the relatively small group of respondents, led to certain conclusion that were not statistically significant: in people with vitamin D3 deficiency, tooth overload, skeletal defects, and tooth crowding were more often observed.

Tooth crowding in the upper and lower arches, and skeletal defects were more often found in correlation with low vitamin D concentration. Skeletal defects occurred in 48.1% of cases. The narrowing of the upper dental arch was found significantly more often in patients with vitamin D deficiency. Crowdings occurred in 47.7% of cases. Moreover, 32.6% of patients with vitamin D deficiency presented for orthodontic treatment, which was a much larger group than that of nondeficit patients. Angle’s Class I, i.e., in the absence of a skeletal defect, was mainly accompanied by an optimal or excessive level of this hormone. The proper concentration of vitamin D3 in the body therefore promotes the proper development of facial bones. This conclusion should be further extended, since there are many more factors that influence bone development, and the examined group of patients was limited. However, current results indicate that there is a need to more thoroughly investigate this topic.

A relatively small number of patients in the study group was diagnosed with skeletal malocclusion. Due to the previously mentioned effect of this hormone on the development and metabolism of bones, including jawbones, and the relationship with, for example, rickets, it would be worthwhile to conduct a study that is fully focused on a group of patients with skeletal defects. As many other factors that were confirmed to influence the development of teeth and bone development, such as persistent organic pollutants (POPs) [25], vitamin D3 plays also a crucial role. Vitamin D modulates tissue contraction, inflammation, and remodeling [26]. Oral supplementation of vitamin D could improve the immune response [27]. Studies concluded that breastfeeding mothers who supplement vitamin D may increase the level of vitamin D in infants; however, there is no evidence to confirm the impact of maternal vitamin D supplementation on radiological rickets in infants [28]. The determination of baseline vitamin D levels and genetic studies of vitamin D receptor (VDR) polymorphisms could be helpful in determining prenatal and postnatal supplementation. This is very important, as increased skeletal defects result in functional disorders, chewing and breathing disorders, and have negative aesthetic, psychological, and social aspects. Their treatment is demanding and multidisciplinary, involving extensive surgical procedures. The current research should be extended in order to compare the degree of vitamin D deficiency and of malocclusion.

## Figures and Tables

**Table 1:** Skeletal and dentoalveolar features; *n*, number of patients.

Feature	*n* (%)
Bite assessment—right side	
● Angle I class	56 (49.1%)
● Angle II class	20 (17.5%)
● Angle III class	14 (12.3%)
● No assessment/not applicable	24 (21.1%)
Bite assessment—right side	
● Canine I class	76 (66.7%)
● Canine II class	23 (20.2%)
● Canine III class	8 (7.0%)
● No assessment/not applicable	7 (6.1%)
Bite assessment—left side	
● Angle I class	53 (46.5%)
● Angle II class	20 (17.5%)
● Angle III class	17 (14.9%)
● No assessment/not applicable	24 (21.1%)
Bite assessment—left side	
● Canine I class	77 (67.5%)
● Canine II class	23 (20.2%)
● Canine III class	8 (7.0%)
● No assessment/not applicable	6 (5.3%)
Malocclusions	
● Distoclussion	10 (8.8%)
● Retrogenia	18 (15.8%)
● Mesioclussion	4 (3.5%)
● Progenia	6 (5.3%)
● Anterior open bite	5 (4.4%)
● Lateral open bite	1 (0.9%)
● Deep bite	23 (20.2%)
● Tete-a-tete	10 (8.8%)
● Crossbite	20 (17.5%)
● Lingual crossbite	2 (1.8%)
● Narrow upper arch	35 (30.7%)
● Widened upper arch	3 (2.6%)
● Shortened upper arch	4 (3.5%)
● Spaced upper arch	14 (12.3%)
● Narrow lower arch	14 (12.3%)
● Widened lower arch	6 (5.3%)
● Shortened lower arch	3 (2.6%)
● Spaced lower arch	8 (7.0%)

**Table 2:** Supplementation of vitamin D.

Feature	Supp. YES *n* = 29	Supp. NO = 85	Y vs. *n* (*p*)
Vitamin D levels (ng/mL)	*n*%	*n*%	
Deficit (up to 30.0 ng/mL)	12, 41.4%	74 87.1%	<0.001
Optimal (above 30.0 mg/mL)	17, 59.6%	11 12.9%	
Vitamin D levels (ng/mL):			
M ± SD	33.36 ± 11.89	20.25 ± 7.47	
Me (Q1; Q3)	33.0 (23.6; 39.9)	19.1 (15.2; 25.5)	0.001
Min—Max	11.5–57.0	7.8–41.9	

Abbreviations: means (M), standard deviations (SD), medians (Me), lower (Q1) and upper (Q3) quartiles, and extreme values: smallest (Min) and largest (Max).

**Table 3:** Comparison of qualitative characteristics of patients differing in vitamin D3 levels and test results.

Risk Factors	Vitamin D Deficiency		Test of Independence	OR (95% PU)
	YES *n* = 86	NO *n* = 28		
Age—up to 28 years	31 (36.0%)	4 (14.3%)	*p* = 0.035	3.38 (1.05; 9.89)
Sex—male	57 (66.3%)	4 (14.3%)	*p* < 0.001	11.8 (3.74; 37.2)
Lack of vitamin D supplementation	74 (86.0%)	10 (35.7%)	*p* < 0.001	11.1 (4.07; 28.6)
Unfavorable factors	57 (66.3%)	17 (60.7%)	*p* = 0.529	1.27 (0.53; 3.06)
Holidays in the sunlight	18 (20.9%)	8 (28.6%)	*p* = 0.388	0.66 (0.25; 1.71)
Vegetarianism	3 (3.5%)	1 (3.6%)	*p* = 1.000	0.98 (0.10; 7.00)
Cardiovascular diseases	3 (3.5%)	2 (7.1%)	*p* = 0.595	0.59 (0.08; 2.64)
Gastrointestinal diseases	6 (7.0%)	4 (14.3%)	*p* = 0.257	0.45 (0.12; 1.65)
Autoimmune disorders	8 (9.3%)	7 (25.0%)	*p* = 0.031	0.31 (0.10; 0.93)
Metabolic disorders	0 (0%)	1 (3.6%)	*p* = 0.246	-
Proper hygienic habits	30 (34.9%)	10 (35.7%)	*p* = 0.936	0.96 (0.40; 2.32)
Teeth overloads	45 (52.3%)	13 (50.0%)	*p* = 0.835	1.10 (0.46; 2.62)
Clinical attachment loss (CAL)	52 (60.5%)	9 (32.1%)	*p* = 0.010	3.23 (1.30; 7.77)
Pathological periodontal pockets	29 (33.7%)	3 (10.7%)	*p* = 0.028	4.24 (1.15; 13.7)
Past orthodontic treatment	28 (32.6%)	8 (28.6%)	*p* = 0.693	1.21 (0.52; 2.84)
Skeletal malocclusions	40 (46.5%)	8 (30.8%)	*p* = 0.142	2.00 (0.78; 4.96)
Crowding	41 (47.7%)	12 (42.9%)	*p* = 0.657	1.21 (0.52; 2.84)
Bruxism	31 (36.1%)	9 (32.1%)	*p* = 0.707	1.19 (0.48; 2.89)

**Table 4:** Comparison of qualitative characteristics of patients differing in vitamin D3 levels and test results.

Risk Factors	Vitamin D Deficiency		OR	OR (95% PU)	
	YES *n* = 86	NO *n* = 28		From	Up to
skeletal malocclusions	28 (32.6%)	6 (21.4%)	1.71	0.59	4.94
crowdings	41 (47.7%)	12 (42.9%)	1.00	0.36	2.75
past orthodontic treatment	28 (32.6%)	8 (28.6%)	1.75	0.62	4.94
malocclusion in Angle class.	28 (32.6%)	6 (21.4%)	1.77	0.65	4.85
narrowed upper arch	31 (36.0%)	3 (10.7%)	4.94	1.38	17.7
widened upper arch	3 (3.5%)	0 (0%)	-	-	-
shortened upper arch	2 (2.3%)	2 (7.1%)	0.32	0.05	2.30
spaced upper arch	13 (15.1%)	1 (3.6%)	4.81	0.60	38.5
narrowed lower arch	11 (12.8%)	3 (10.7%)	1.22	0.32	4.74
widened lower arch	6 (7.0%)	0 (0%)	-	-	-
shortened lower arch	1 (1.2%)	2 (7.1%)	0.15	0.01	1.76
spaced lower arch	5 (5.8%)	3 (10.7%)	0.51	0.11	2.31
crowdings- upper arch	32 (37.2%)	3 (10.7%)	4.94	1.38	17.7
crowdings- lower arch	39 (45.3%)	11 (39.3%)	1.28	0.54	3.06
crossbite	18 (20.9%)	2 (7.1%)	6.16	1.32	28.8
lingual crossbite	2 (2.3%)	0 (0%)	-	-	-
anterior open bite	3 (3.5%)	2 (7.1%)	0.47	0.07	2.97
lateral open bite	1 (1.2%)	0 (0%)	-	-	-
deep bite	19 (22.1%)	4 (14.3%)	1.70	0.53	5.51
mesiocclusion	4 (4.7%)	0 (0%)	-	-	-
progenia	5 (5.8%)	1 (3.6%)	1.67	0.19	14.9
tete-a-tete	9 (10.5%)	1 (3.6%)	3.16	0.38	26.1
distoclussion	7 (8.1%)	3 (10.7%)	0.74	0.18	3.07
retrogenia	15 (17.4%)	3 (10.7%)	1.76	0.47	6.60

## Data Availability

Datasets generated and/or analyzed during the current study are available from the corresponding author on reasonable request.
